# Thymic resident NKT cell subsets show differential requirements for CD28 co-stimulation during antigenic activation

**DOI:** 10.1038/s41598-020-65129-3

**Published:** 2020-05-19

**Authors:** Susannah C. Shissler, Nevil J. Singh, Tonya J. Webb

**Affiliations:** Department of Microbiology and Immunology and the Marlene and Stewart Greenebaum Comprehensive Cancer Center, University of Maryland School of Medicine, Baltimore, MD 21201 USA

**Keywords:** Lymphocyte activation, NKT cells

## Abstract

Natural killer T (NKT) cells rapidly respond to antigenic stimulation with cytokine production and direct cytotoxicity. These innate-like characteristics arise from their differentiation into mature effector cells during thymic development. A subset of mature NKT cells remain thymic resident, but their activation and function remain poorly understood. We examined the roles of CD28 and CTLA-4 in driving the activation of thymic resident NKT cells. In contrast to studies with peripheral NKT cells, the proliferation of thymic NKT cells was significantly impaired when CD28 engagement was blocked, but unaffected by CTLA-4 activation or blockade. Within NKT subsets, however, stage 3 NKT cells, marked by higher NK1.1 expression, were significantly more sensitive to the loss of CD28 signals compared to NK1.1− stage 2 NKT cells. In good agreement, CD28 blockade suppressed NKT cell cytokine secretion, lowering the ratio of IFN-γ:IL-4 production by NK1.1+ NKT cells. Intriguingly, the activation-dependent upregulation of the master transcription factor PLZF did not require CD28-costimulation in either of the thymic NKT subsets, underlining a dichotomy between requirements for early activation vs subsequent proliferation and effector function by these cells. Collectively, our studies demonstrate the ability of CD28 co-stimulation to fine tune subset-specific responses by thymic resident NKT cells and contextually shape the milieu in this primary lymphoid organ.

## Introduction

Natural killer T (NKT) cells are a subset of innate-like T cells, that possess a T cell receptor (TCR) and develop in the thymus^1^. In contrast to classic T cells, which recognize peptide antigen in the context of the antigen presenting molecules MHC class I or II, NKT cells recognize glycolipid antigens presented by the non-polymorphic, MHC class Ib molecule, CD1d^2^. NKT cells can be subdivided into distinct subsets, type I (invariant) and type II (diverse), based on their TCR^3,4^. Type I NKT cells recognize the canonical ligand α-Galactosylceramide (α-GalCer) and most possess a semi-invariant TCR containing the Vα14Jα18 chain rearrangement paired with β chains of limited diversity^5,6^. As innate-like lymphocytes, NKT cells differentiate into mature effector cells during thymic development^7–10^. Therefore, type I NKT cells can be further divided into 3 subtypes that mirror the T helper subtypes including NKT1, NKT2, and NKT17^11^. These subsets are primarily identified by differences in the levels of PLZF expression^12^ following differentiation signals in the thymus including TCR engagement^13^. In C57BL/6 mice, this hierarchy is defined by the levels of NK1.1 and PLZF as, NK1.1+ stage 3 NKT cells correspond to NKT1 and NK1.1- stage 2 NKT cells encompass NKT2 and NKT17^10,12,14^. Recent studies have indicated that in addition to developing in the thymus, a proportion of mature NKT cells maintain residency in the thymus where they impact the thymic environment; thus, the thymus is an extremely important immunological niche for NKT cells^12,15^.

Similar to T cells, engagement of costimulatory molecules affects NKT cell activation and function. In the canonical CD28 axis, B7 molecules (CD80/CD86) expressed by antigen presenting cells interact with CD28 on T cells leading to the delivery of an activating signal which promotes the proliferation, survival and differentiation of T cells. CTLA-4 either limits co-stimulation by competing with CD28 for B7-engagement or delivering a negative co-inhibitory signal^16^. CD28 is constitutively expressed on NKT cells^17,18^. Thymic NKT cell populations are decreased with KO of CD28 or CD80/86^19,20^ and with overexpression of CD28 or CD86^21^. Interestingly, thymic NK1.1+ NKT cells were the most significantly affected^20,21^. Although peripheral populations may be reduced initially, they appear to normalize with age, indicating CD28 may not be involved in homeostatic proliferation^19,21^. Nevertheless, blockade or KO of CD28 or CD80/86 reduces proliferation^5,20^, and production of IFN-γ and IL-4 by these cells, with a stronger effect on IL-4 production^17,19,20^. Blockade of PD-1 in CD28 KO mice enhances IFN-γ secretion, but not to WT levels^19^. Concordantly, ex vivo stimulation and expansion of primary human or murine NKT cells using artificial antigen presenting cells is enhanced by inclusion of anti-CD28 mAb^18^. CTLA-4 ligation typically results in co-inhibitory signals, but a study from the Taniguchi lab indicated that CTLA-4 blockade of NKT cells from Vα14-Tg mice inhibited NKT cell proliferation^5^. Taken together, although the consequence of CD28 and CTLA-4 engagement on subsets of type 1 NKT cells during activation, remain unclear, it is generally believed that costimulation with CD28 is supportive, but not required for thymic NKT cell development.

Here, we find that thymic NKT cells have subset-specific requirements for CD28 stimulation which are limited to the proliferative phase of NKT cell activation but not upregulation of PLZF. We probed the CD28 axis using CD80/86 blockade or CTLA-4 ligation with an optimized *ex vivo* thymic NKT proliferation assay. We found that while co-inhibitory signals from CTLA-4 do not significantly affect NKT cell activation and proliferation, CD80/86 blockade differentially impacts distinct stages of NKT cells. While proliferation of both stage 2 and stage 3 NKT cells is decreased by CD28 blockade, inhibition of CD28 also restrained more stage 3 NKT cells in the undivided population. PLZF was upregulated in undivided NK1.1- NKT cells despite CD28 blockade. Additionally, stage 2 NKT cells were responsive to lower concentrations of antigen than stage 3. Finally, cytokine production was significantly decreased by CD28 blockade and decreased antigen concentration – reducing the ratio of IFN-γ:IL-4 production and mirroring changes in proliferation. Collectively, these data indicate that CD28 signals play a role in thymic type 1 NKT cells, distinct from that previously observed for bulk peripheral NKT cells.

## Results

### Enrichment of mature thymic NKT cells by negative selection maintains subset composition and phenotype

**Figure 1:**
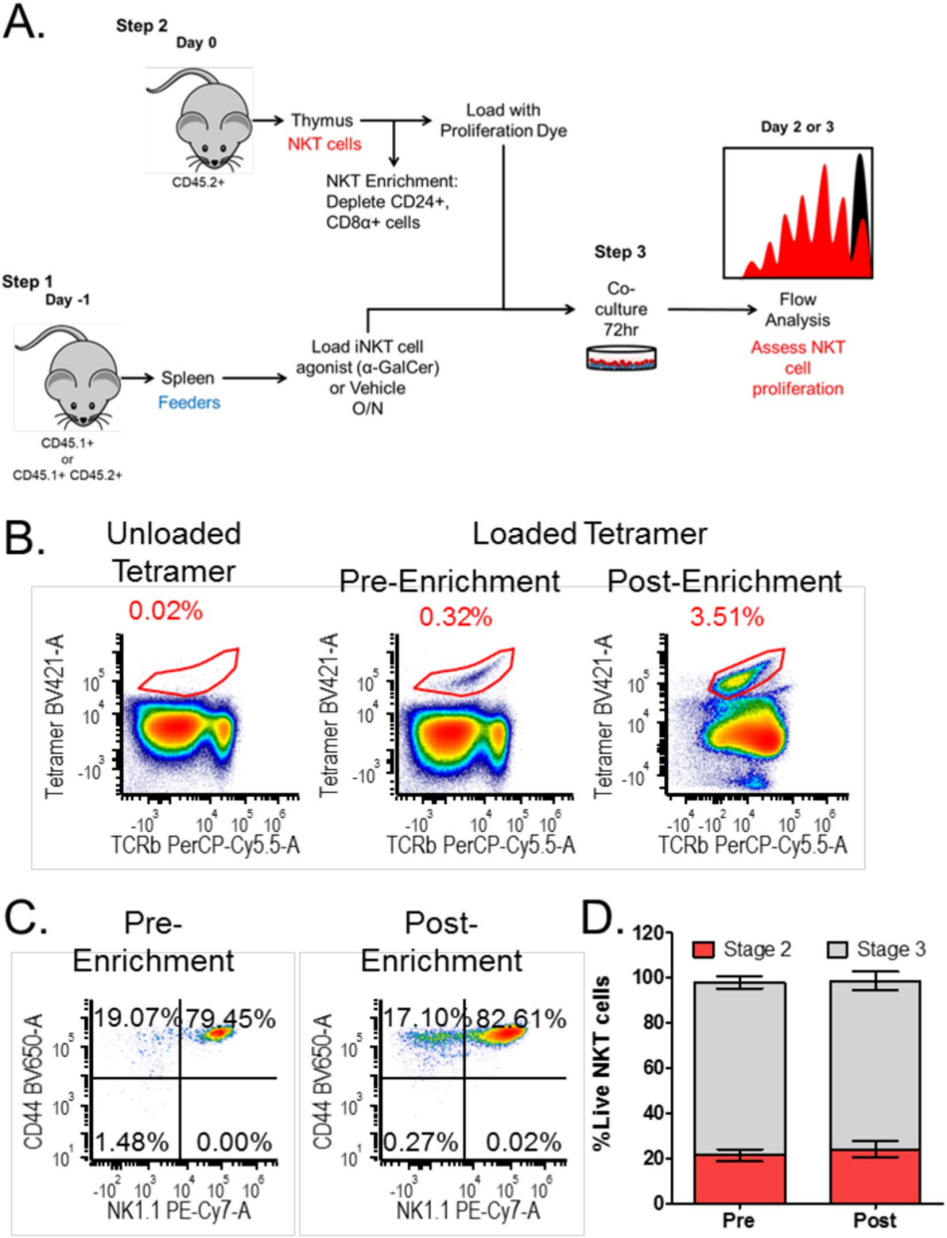
Depletion of CD8α and CD24 enriches for mature thymic NKT cells without altering their composition. (**A**) Schematic of the thymic NKT cell proliferation assay. (**B**) NKT cell populations (αGC:CD1d tetramer+TCRβ+) pre- and post-enrichment with unloaded tetramer shown as a control. (**C**) Pre- and post-enrichment NKT cell populations subdivided into stage 2 (CD44 + NK1.1−) and stage 3 (CD44 + NK1.1+). (**D**) The percentage of stage 2 and stage 3 NKT cells pre- and post-enrichment. Relevant statistical analyses are discussed in the text. Data correspond to mean+/− SEM of 3 biological replicates. Statistical significance determined by student’s t test. Flow cytometry gating strategy is outlined in the Materials and Methods.

Type I NKT cells typically make up 0.2–1.5% of thymic lymphocytes and can be subdivided into multiple fractions, due to expression of specific cell surface markers and transcription factors^12^. The functional response of these fully mature cells to a stimulatory antigen has not been well characterized. To obtain a substantial number of NKT cells, enrichment is necessary (Fig. 1A). Prior literature examining distinct populations of NKT cells utilized fluorescence activated cell sorting (FACS) prior to stimulation^22,23^. Such approaches involving positive labeling of NKT cells is confounded by modification of T and NK cell markers and their activated, effector phenotype. In addition to potentially inducing activation, positive selection using the TCR has been shown to skew NKT cell subsets towards NKT2^24^. Instead, negative selection by depletion of CD24+ and CD8+ thymocytes enriches NKT cells ~10 fold (Fig. 1B). This method specifically enriches mature thymic NKT cell populations because it will deplete NKT cells undergoing positive selection (which express CD8) and stage 0 NKT cells (which express CD24). Importantly, depletion of CD8 and CD24 does not significantly alter the proportion of stage 2 and stage 3 NKT cells (Fig. 1C,D). These data agree with a recently published protocol for NKT enrichment by CD24 depletion^24^. These enriched cells can then be labeled with a proliferation dye, such as CFSE or Cell Trace Violet (Fig. 1A) for further analysis.

**Figure 2:**
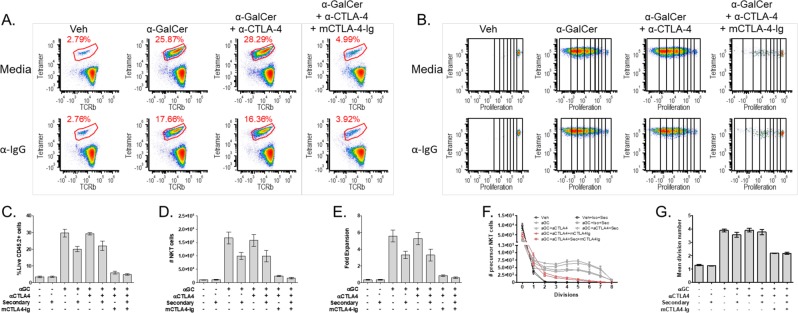
NKT cell activation is not affected by CTLA-4 blockade or activation, but is inhibited by CD80/86 blockade. (**A**) Representative plots of NKT cell populations (αGC:CD1d tetramer+TCRβ+) after 3-day co-culture. (**B**) Representative plots of NKT cell population proliferation with division bins denoted after 3-day co-culture. (**C**) NKT cell percentage (**D**) Total NKT cell number, and (**E**) NKT cell fold expansion post stimulation. (**F**) The precursor proliferation curve. (**G**) The mean division number of proliferating precursors. All data displayed in graphs correspond to mean +/− SEM of 3 biological replicates. Statistical significance was determined using one-way ANOVA followed by Bonferroni tests. Relevant statistical analyses are discussed in the text. Flow cytometry gating strategy outlined in Materials and Methods.

In order to assess thymic NKT cell responses to antigenic stimulation, we used syngeneic splenocytes as feeder cells (Fig. 1A), which provides a more physiological stimulation, compared to plate-bound or bead-based stimulations. To ensure that the target cells can be differentiated from the feeder cells, we used congenic CD45 markers (NKT cells derived from CD45.2 + B6 mice, while the feeder cells were F1s expressing both CD45.1 and CD45.2). Splenocytes were loaded with antigen (α-GalCer) or vehicle control overnight. Enriched thymic NKT cells and antigen-loaded feeder cells were co-cultured at a 1:1 ratio (Fig. 1A). By 48 hours, clusters of proliferating cells were visible under a light microscope in the wells containing α-GalCer-loaded splenocytes (data not shown). Co-cultures were maintained 72 hours and then harvested for flow cytometric analysis to assess proliferation (Fig. 1A). Cells harvested at 48 hours will have completed up to 5 rounds of expansion (data not shown), whereas cells harvested at 72 hours will have reached up to 8 divisions (Fig. 2B).

### Thymic NKT cell proliferation is CD28 dependent and not inhibited by CTLA-4

We used a panel of monoclonal antibodies in conjunction with the proliferation assay described above to characterize the effects of CTLA-4 blockade on thymic NKT cells. Representative flow plots of the NKT cell population after the 3-day co-culture are displayed in Fig. 2A. As expected, the addition of α-GalCer significantly increased the percentage of NKT cells (8.6 fold, Veh to α-GalCer; p < 0.0001) (Fig. 2C) as well as their absolute number (16.7 fold, Veh to α-GalCer; p < 0.0001) (Fig. 2D). We then combined this system with a blocking antibody against CTLA-4^16^. Blocking CTLA-4 by addition of excess α-CTLA-4 did not significantly alter NKT cell expansion, neither the percentage (Fig. 2C) nor absolute cell number (Fig. 2D) (α-GalCer to α-GalCer+α-CTLA-4; n.s.). We then asked if triggering inhibitory signals via CTLA-4 could impair NKT cell proliferation. Towards this end, we used an approach similar to a previously validated method to trigger CTLA-4 by crosslinking^25^. CTLA-4 activation by the addition of α-CTLA-4 and a crosslinking secondary Ab (α-Hamster IgG) did not significantly impact NKT cell growth (Fig. 2C, percentages or Fig. 2D, absolute numbers) compared to control (α-GalCer+Iso+secondary to α-GalCer+α-CTLA-4+secondary; n.s.). NKT cell fold expansion (Fig. 2E) was calculated by comparing the total number of thymic NKT cells plated to the total number of thymic NKT cells harvested after the 3-day co-culture. Stimulation with α-GalCer resulted in a significantly higher fold expansion (5.5 fold ± 1.2, Veh to α-GalCer; p < 0.0001) and was not affected by blocking or activating CTLA-4 (Fig. 2E, α-GalCer to α-GalCer±Iso±α-CTLA-4±secondary; n.s.). Taken together, these data indicate that thymic NKT cells are resistant to CTLA-4 blockade or ligation with regard to the control of their proliferative burst. Importantly, as discussed in the introduction, these results differ from prior literature on peripheral (splenic) NKT cells from Vα14Tg mice^5^, suggesting that thymic NKT cells derived from unmanipulated C57BL/6 mice behave quite differently.

The lack of a direct consequence of CTLA-4, now allowed us to examine the requirement for CD28 co-stimulation using a reagent (soluble mouse CTLA-4-Ig) that binds CD80/86 with high affinity and prevents both CD28 and CTLA-4 engagement^26^ and therefore inhibits T cell proliferation. Treatment with mCTLA-4-Ig abrogated the proliferative burst typically triggered by α-GalCer from NKT cells. These NKT cells failed to expand - as observed by percentage (Fig. 2C), absolute cell number (Fig. 2D), and fold expansion (Fig. 2E) (Veh to α-GalCer+mCTLA-4-Ig+α-CTLA-4±Secondary; n.s.). Importantly, we performed the CD80/86 blockade in conjunction with α-CTLA-4 with or without the secondary Ab. Given that no effects are observed by treatment with α-CTLA-4 in the presence or absence of the secondary Ab, we can conclude that the suppression observed in the mCTLA-4-Ig treatments is due to the prevention of CD28 signaling.

In the case of conventional αβ T cells, an extensive literature has precisely quantitated the contribution of CD28 signals to proliferation, cytokine production and differentiation^27–29^. To extract similar quantitative parameters from the NKT proliferation assay, we used a previously published methodology^27^. As shown in Fig. 2F, 65% of the NKT cells entered proliferation after addition of α-GalCer (absolute number of undivided NKT cells in Veh and α-GalCer is 8496 ± 2171 vs 2933 ± 608; p < 0.001). These dividing cells also proliferated more, marked by a shift of the curve to the right (Fig. 2F) and evident in the mean division number (3.88 ± 0.2, Veh to α-GalCer; p < 0.0001) (Fig. 2G). CD28 blockade drastically shifted the division curve of seeded cells back towards the vehicle-stimulated NKT cells (Fig. 2F) and significantly decreased their mean division number (2.18 ± 0.02, α-GalCer±Iso±α-CTLA-4±secondary to α-GalCer+mCTLA-4-Ig+α-CTLA-4±secondary; p < 0.0001) (Fig. 2G). However, NKT cells under CD28 blockade still proliferated significantly more than vehicle-treated NKT cells (Veh to α-GalCer+mCTLA-4-Ig+α-CTLA-4±secondary; p < 0.001) (Fig. 2G). Reduced division and expansion under CD28 blockade after activation correlates with prior literature citing decreased BrdU incorporation of *in vivo*, thymic, developing NK1.1 + T cells in CD80/86 KO mice^20^, indicating that CD28 is important for proliferation during development and activation.

### Subtypes of thymic Type 1 NKT cells are differentially affected by CD80/86 blockade

**Figure 3:**
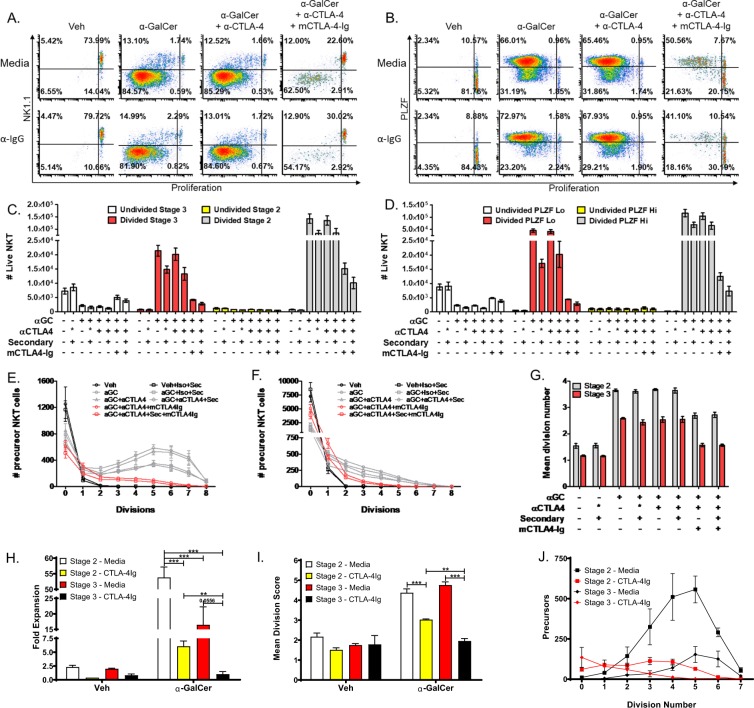
NKT cell subtypes are differentially affected by CD80/86 blockade. (**A**) Representative plots of NKT cell populations stratified by NK1.1 expression and proliferation dye dilution. Quadrants represent the following populations: UR, Undivided Stage 3, UL, Divided Stage 3, LR, Undivided Stage 2, LL, Divided Stage 2. (**B**) Representative plots of NKT cell populations stratified by PLZF expression and proliferation dye dilution. Quadrants represent the following populations: UR, Undivided PLZF-Hi, UL, Divided PLZF-Hi, LR, Undivided PLZF-Lo, LL, Divided PLZF-Lo. (**C**) The number of NKT cells present in the following bins: Undivided Stage 3 (white bars), Divided Stage 3 (red bars), Undivided Stage 2 (yellow bars), Divided Stage 2 (grey bars). (**D**) The number of NKT cells present in the following bins: Undivided PLZF-Hi (white bars), Divided PLZF-Hi (red bars), Undivided PLZF-Lo (yellow bars), Divided PLZF-Lo (grey bars). (**E**) The precursor proliferation curve of stage 2 NKT cells. (**F**) The precursor proliferation curve of stage 3 NKT cells. (**G**) The mean division number of stage 2 and stage 3 precursor cells. (**H**) Fold expansion of FACS-isolated stage 2 and stage 3 NKT cells after co-culture with splenocytes loaded with 10 ng/mL α-GalCer. (**I**) Mean division score of sorted stage 2 and stage 3 NKT cells after co-culture with splenocytes loaded with 10 ng/mL α-GalCer. (**J**) Precursor proliferation curve of sorted NKT cells after co-culture with splenocytes loaded with 10 ng/mL α-GalCer. All data displayed in graphs correspond to mean +/− SEM of 3 biological replicates. Statistical significance was determined using one-way ANOVA followed by Bonferroni tests. Relevant statistical analyses are discussed in the text. Flow cytometry gating strategy outlined in Materials and Methods.

Originally thought to delineate mature from immature NKT cells, NK1.1 expression defines stage 3 NKT cells that have acquired the ability to predominantly produce IFN-γ^10^. It was previously reported that the development of stage 3 NKT cells is more significantly reduced by CD28 or CD80/86 KO than stage 2 NKT cells^20,21^. Unstimulated thymic type 1 NKT cells from 5–6-week-old C57BL/6 mice are approximately 80% stage 3 (NK1.1+) and 20% stage 2 (NK1.1−) (Fig. 1D), but the proportion inverts to become 15% stage 3 and 85% stage 2 (Fig. 3A) after stimulation. α-GalCer-stimulated stage 2 NKT cells (Fig. 3C) were not significantly altered in the undivided bin (Veh to α-GalCer; n.s.) but were significantly increased in the divided bin (Veh to α-GalCer; p < 0.0001). Conversely, stage 3 NKT cells were significantly decreased (Veh to α-GalCer; p < 0.001) (Fig. 3C) in the undivided bin and increased (Veh to α-GalCer; p < 0.0001) in the divided bin. Concordantly, both stage 2 and 3 NKT cells had precursor proliferation curves (Fig. 3E,F) that shifted to the right of vehicle-stimulated NKT cells and significantly higher mean division numbers, 3.65 ± 0.09 compared to 2.58 ± 0.05, for stage 2 and stage 3, respectively (Veh to α-GalCer; p < 0.0001) (Fig. 3G).

In agreement with our conclusions above, CTLA-4 blockade or activation did not alter the proliferation of either stage 2 or stage 3 NKT cells (Fig. 3A,C,E–G) (stage 2 and 3 mean division numbers, undivided populations, and divided populations for α-GalCer to α-GalCer+αCTLA-4 and α-GalCer+Iso+secondary to α-GalCer+αCTLA-4+secondary; n.s.). Conversely, CD28 blockade negated both the decrease in undivided and increase in divided stage 3 NKT cells following α-GalCer stimulation (Fig. 3C) (Veh±Iso±secondary to α-GalCer+mCTLA-4-Ig+α-CTLA-4±secondary; n.s.). CD28 blockade significantly reduced the expansion of activated stage 3 precursor cells, as demonstrated by a shift to the left of the precursor proliferation curve (Fig. 3F) and a mean division number of only 1.6 ± 0.1 (α-GalCer±Iso±α-CTLA-4±secondary to α-GalCer+mCTLA-4-Ig+α-CTLA-4±secondary; p < 0.0001) (Fig. 3G). Concomitantly, CD28 blockade significantly reduced the number of divided stage 2 NKT cells (Fig. 3C) observed after α-GalCer stimulation (α-GalCer+α-CTLA-4 to α-GalCer+mCTLA-4-Ig+α-CTLA-4; p < 0.0001) and decreased the expansion of stage 2 precursors, as indicated by a shift of the precursor proliferation curve to the left and a reduced mean division score of 2.69 ± 0.17 (α-GalCer±Iso±α-CTLA-4±secondary to α-GalCer+mCTLA-4-Ig+α-CTLA-4±secondary; p < 0.0001) (Fig. 3E,G).

Analysis of CFSE dilution and NK1.1 expression indicates that α-GalCer-stimulated stage 2 NKT cells expand more efficiently than stage 3 NKT cells. α-GalCer-stimulated stage 2 NKT cells consistently had significantly higher mean division numbers (Fig. 3G) than stage 3 NKT cells (3.65 ± 0.09 vs 2.58 ± 0.04, stage 2 α-GalCer±Iso±α-CTLA-4±secondary±m-CTLA-4-Ig to stage 3 α-GalCer±Iso±α-CTLA-4±secondary±m-CTLA-4-Ig; p < 0.0001). Interestingly, 70% of stage 3 NKT cells (undivided precursors number 2179 and 7271 for α-GalCer and Veh, respectively) committed to proliferation after α-GalCer stimulation compared to only 37% of stage 2 NKT cells (undivided precursors number 793 and 1253 for α-GalCer and Veh, respectively) (Fig. 3E,F). Under CD28 blockade, the percentage of stage 3 NKT cells recruited to proliferation decreased to 30% (undivided precursors number 5067 and 7271 for α-GalCer+α-CTLA-4 + m-CTLA-4-Ig and Veh, respectively) whereas the percentage of stage 2 NKT cells recruited increases to 51% (undivided precursors are 619 vs 1253 for α-GalCer+α-CTLA-4 + m-CTLA-4-Ig and Veh, respectively). It is known that NKT cell responses are altered by the type and concentration of stimulus used^22^. According to our results, blockade of CD28 decreases the expansion of activated stage 2 and 3 NKT cells and decreases the number of stage 3 cells achieving first division. These results align with decreased development of NK1.1+ NKT cells in CD80/86 KO mice^20,21^.

Stage 3 NKT cells are predominantly NKT1, whereas stage 2 NKT cells are composed of NKT2 and NKT17^12^. Since earlier studies evaluating the CD28 costimulatory pathway in NKT cells were published prior to the identification of these effector subtypes, the differential effects of CD28 on these NKT cell subsets has not yet been reported. As discussed above, PLZF expression is helpful in identifying NKT cell effector subtypes (Fig. 3B), with PLZF-Hi corresponding to NKT2/17 and PLZF-Lo corresponding to NKT1 cells. Stimulation with α-GalCer inverted the ratio of PLZF-Lo: PLZF-Hi cells –from 85:15 to 30:70 (Fig. 3B,D). As with stage 2 NKT cells, PLZF-Hi cells expanded more efficiently with a higher total cell number (Fig. 3D), and more cells reaching 8 divisions (Fig. 3B). Blocking CD28 costimulation significantly decreased the number of divided PLZF-Lo cells (α-GalCer to α-GalCer+mCTLA-4-Ig+α-CTLA-4±secondary; p < 0.0001) (Fig. 3D). Similarly, the number of PLZF-Hi divided cells was significantly decreased (α-GalCer to α-GalCer+mCTLA-4-Ig+α-CTLA-4±secondary; p < 0.0001), indicating decreased expansion (Fig. 3D). In alignment with decreased IFN-γ and IL-4 production observed in previous literature^17,19^, our proliferation data demonstrates that both NKT1 and NKT2 cells are adversely affected by CD80/86 blockade. Prior studies have indicated that stage 3 NKT cells can lose NK1.1 after activation^33^. In order to ensure that the stark differences observed between stage 2 and stage 3 NKT cell proliferation ex vivo were not due to collapse of the stage 3 population into the stage 2 population, we used FACS-isolated stage 2 and stage 3 NKT cells (see Supplementary Fig. 1 online) in the co-culture system. We found that stage 3 NKT cells downregulate NK1.1 during proliferation – progressing from NK1.1+ in the undivided population to NK1.1- by division 3–4. Despite the convergence of stage 3 and stage 2 NKT cells, analysis of the fold expansion (Fig. 3H), mean division score (Fig. 3I), and precursor proliferation curve (Fig. 3J) reiterated the results of the bulk stimulation experiments, and suggest that the mostly likely interpretation of our data is that stage 3 NKT cells proliferate less and are more sensitive to CD28 blockade. Stage 3 NKT cells expanded significantly less than stage 2 NKT cells after α-GalCer stimulation (16.5 vs 53.8-fold, p < 0.0001, Fig. 3H). Blockade of CD28 significantly reduced the expansion (53.8 vs 6.1-fold, p < 0.0001, Fig. 3H) and mean division score (4.4 vs 3.0, p < 0.0001, Fig. 3I) of stage 2 NKT cells and shifted the precursor proliferation graph to the left (Fig. 3J). Similarly, CD28 blockade of stage 3 NKT cells reduced their expansion (16.5 vs. 1.0, p = 0.0556, Fig. 3H) and mean division number (4.8 vs 2.0, p < 0.0001, Fig. 3I). Importantly, the fold expansion (1.0 vs 6.1, p < 0.001, Fig. 3H) and mean division number (2.0 vs 3.0, p < 0.001, Fig. 3I) of stage 3 NKT cells under CD28 blockade were significantly lower than stage 2 NKT cells under CD28 blockade – confirming that the proliferation of stage 3 NKT cells is more significantly impacted by CD28 blockade.

### PLZF is upregulated prior to division while CD28 is upregulated on dividing cells

**Figure 4:**
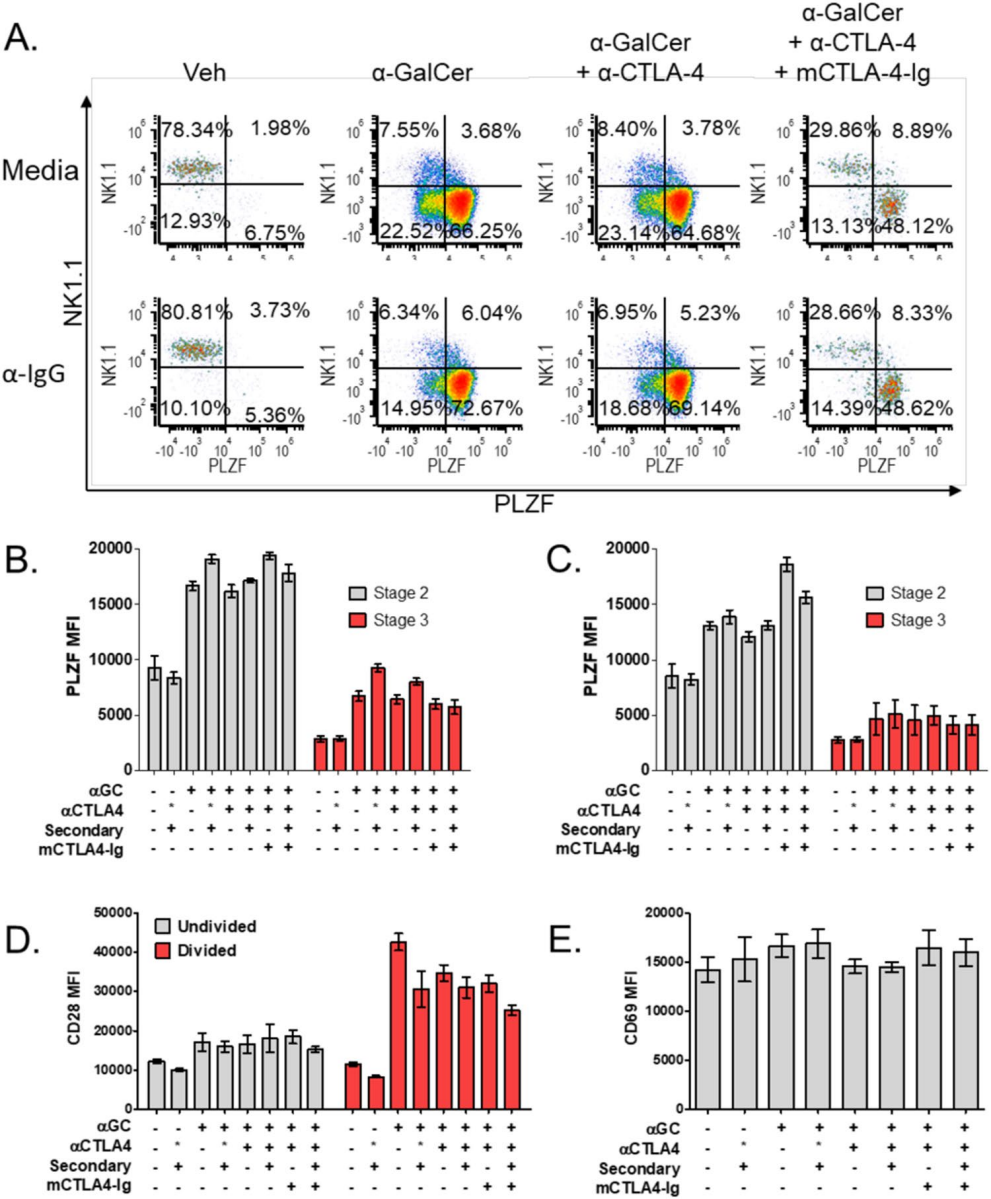
PLZF and CD28 expression are altered following stimulation. (**A**) Representative plots of NKT cell populations stratified by NK1.1 and PLZF expression. Quadrants represent the following populations: U, Stage 3, NK1.1 + , L, Stage 2, NK1.1- (**B**) The MFI of PLZF in bulk stage 2 (grey bars) and stage 3 (red bars) NKT cells. (**C**) The MFI of PLZF in undivided stage 2 (grey bars) and stage 3 (red bars) NKT cells. (**D**) The MFI of CD28 on undivided (red bars) and divided (grey bars) NKT cells. (**E**) The MFI of CD69 on NKT cells. All data displayed in graphs correspond to mean +/− SEM of 3 biological replicates. Statistical significance was determined using one-way ANOVA followed by Bonferroni tests. Relevant statistical analyses are discussed in the text. Flow cytometry gating strategy outlined in Materials and Methods.

PLZF expression in homeostatic conditions is strongly correlated with NKT cell effector type ^10,12,14^, but PLZF expression after stimulation is less extensively characterized. Our curiosity was piqued by the observation that the number (Fig. 3C,D) and percentage (Fig. 3A,B) of PLZF-Lo NKT cells was approximately twice that of NK1.1 + , stage 3 NKT cells. Indeed, examination of NK1.1 and PLZF expression indicated the presence of PLZF-Lo, NK1.1- cells (Fig. 4A) in α-GalCer-stimulated NKT cell populations, signifying the plasticity of stimulated, mature NKT cells. Consequently, we analyzed PLZF expression in stage 2 and stage 3 NKT cells (Fig. 4B) and found that stimulation with α-GalCer significantly increased the expression of PLZF in all groups (Veh±Iso±secondary to α-GalCer±Iso±α-CTLA-4±secondary; p < 0.0001). CD28 blockade did not affect PLZF upregulation with α-GalCer stimulation (Veh±Iso±secondary to α-GalCer+CTLA-4-Ig+α-CTLA-4±secondary; p < 0.0001 stage 2 and p < 0.001 stage 3). When we assessed PLZF expression in undivided stage 2 and 3 NKT cells (Fig. 4C), we found that undivided, α-GalCer-stimulated, stage 2 NKT cells have a significant increase in PLZF expression (Veh to α-GalCer+α-CTLA-4; p < 0.05) that is further increased under CD28 blockade (α-GalCer+α-CTLA-4+mCTLA-4-Ig; p < 0.0001). Undivided stage 3 NKT cells trend toward higher PLZF expression, but the difference is not significant (Fig. 4C). The observation that PLZF is still upregulated in undivided cells under CD28 blockade suggests that PLZF upregulation is TCR-dependent and CD28-independent. We propose that TCR signaling induces PLZF upregulation in anticipation of proliferation, but the lack of CD28 signaling keeps these cells in an undivided state, causing PLZF levels to be even higher under CD28 blockade.

CD28 expression on both undivided and divided cell populations was assessed to examine the differential effects of CD80/86 blockade on NKT cell subtypes (Fig. 4D). Our finding that CD28 is constitutively expressed on thymic NKT cells correlates with prior literature indicating the CD28 KO negatively impacts thymic NKT cells ^17,18^. Our data indicates that CD28 expression was significantly increased on α-GalCer-stimulated, actively dividing NKT cells (Veh to α-GalCer; p < 0.0001) (Fig. 4D). This increase is specific to activated, dividing cells and did not decrease with either CTLA-4 activation/blockade or CD80/86 blockade (α-GalCer to α-GalCer+α-CTLA-4±secondary±mCTLA-4-Ig; n.s.) (Fig. 4D). Increased CD28 expression during proliferation indicates a heightened requirement for CD28 stimulation during this time as evidenced by the decreased mean division number of both stage 2 and 3 dividing precursors (Fig. 3G) in the presence of CD80/86 blockade. This correlates well with CD28 function in conventional T cells where it is known to enhance survival and proliferation after antigenic stimulation^29,30^. Although prior reports have indicated CD69 upregulation after activation^31^, our analysis found CD69 to be highly expressed on all NKT cells and unchanged by stimulation (Fig. 4E) even when we assessed dividing and undivided populations (data not shown). Given that our time course was 3 days, our data does not rule out changes in CD69 at earlier time points after stimulation.

### α-GalCer-induced proliferation of stage 3 NKT cells is inhibited by CD28 blockade in vivo

**Figure 5:**
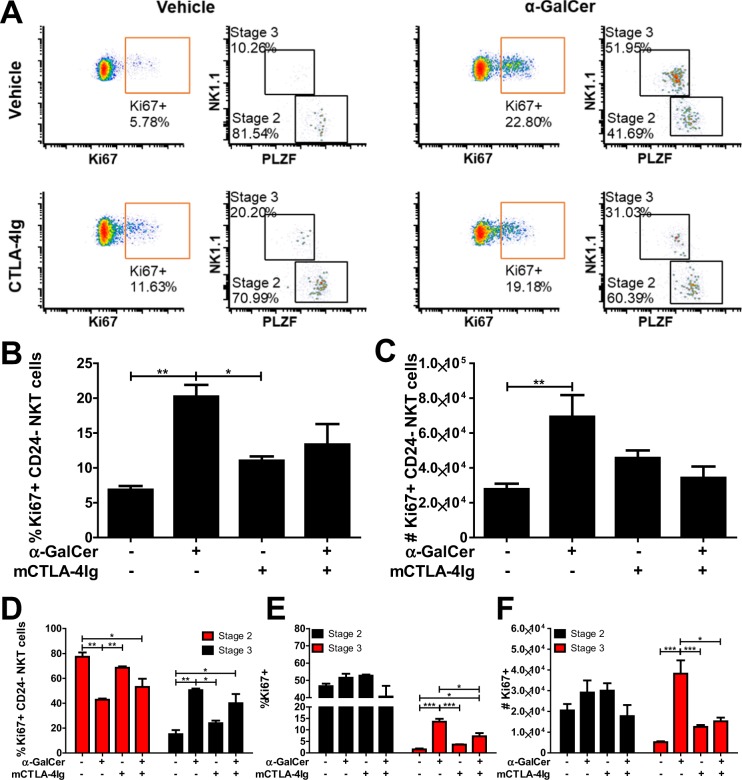
α-GalCer-induced proliferation of stage 3 NKT cells is inhibited by CD28 blockade *in vivo*. Mice received vehicle or 50 μg CTLA-4Ig IP and then vehicle or 2 μg α-GalCer IV 1 hour later. Mice were euthanized for analysis at 48 hour post α-GalCer administration. (**A**) Representative plots of Ki67 expression by NKT cells and the stratification of Ki67+ NKT cells by NK1.1 and PLZF expression. (**B**) The percentage of Ki67+ NKT cells. (**C**) The number of Ki67+ NKT cells. (**C**) The percent of Ki67+ NKT cells that are stage 2 and stage 3. (**D**) The percent of stage 2 and stage 3 cells that are Ki67+. (**E**) The number of stage 2 and stage 3 cells that are Ki67+. All data displayed in graphs correspond to mean +/− SEM of 3 biological replicates. Statistical significance was determined using one-way ANOVA followed by Bonferroni tests. P values as follows: *p < 0.05, **p < 0.001, and ***p < 0.0001. Flow cytometry gating strategy outlined in Materials and Methods.

In accordance with our ex vivo results, prior studies have indicated an increase in the proportion of stage 2 NKT cells after intrathymic α-GalCer injection and decreased baseline stage 3 proliferation in CD28KO mice^20,32^. In order to determine whether the stark differences observed ex vivo were recapitulated in vivo, we administered vehicle or α-GalCer to mice in the presence or absence of CD28 blockade (CTLA-4Ig). At 48 hours post-stimulation, thymic NKT cells were assessed for proliferation by Ki67 expression (Fig. 5A). Compared to vehicle stimulation, α-GalCer administration significantly increased the percentage (p < 0.001, Fig. 5B) and absolute number (p < 0.001, Fig. 5C) of NKT cells expressing Ki67. Of Ki67+ NKT cells, 80% were stage 2 at baseline (Fig. 5D). However, upon α-GalCer administration, the proportion stage 3 significantly increased (p < 0.001) and stage 2 significantly decreased (p < 0.001) within Ki67+ NKT cells. Approximately 50% of stage 2 NKT cells expressed Ki67 at baseline (Fig. 5E), whereas approximately 1.5% of stage 3 NKT cells expressed Ki67 at baseline. While stimulation with α-GalCer did not alter the percentage or absolute number of Ki67+ stage 2 NKT cells, α-GalCer stimulation did significantly increase the percentage (13.5%, p < 0.0001, Fig. 5E) and absolute number (3.8 × 10^4^, p < 0.0001, Fig. 5F) of Ki67+ stage 3 NKT cells compared to vehicle. α-GalCer stimulation in the presence of CD28 blockade significantly decreased the percentage (7%, p < 0.05, Fig. 5E) and absolute number (1.5 × 10^4^, p < 0.05, Fig. 5E) of Ki67+ stage 3 NKT cells compared to α-GalCer stimulated. Cumulatively, CD28 blockade significantly decreased the recruitment of stage 3, but not stage 2, NKT cells to proliferation after α-GalCer administration – recapitulating the ex vivo results.

### Stage 3 NKT cells are more sensitive to CD28 blockade than Stage 2 NKT cells

**Figure 6:**
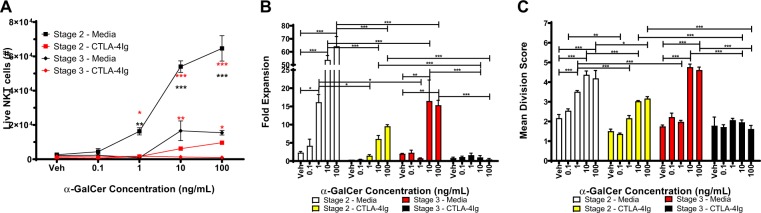
Stage 3 NKT cells are more sensitive to CD28 blockade than Stage 2 NKT cells. (**A**) Number of live stage 2 and stage 3 NKT cells after co-culture with splenocytes loaded with 0.1-100 ng/mL α-GalCer. Black asterisks compare stage 2 to stage 3 while red asterisks compare media to CTLA-4Ig within stage 2 or stage 3. (**B**) Fold expansion of NKT cells after co-culture with splenocytes loaded with 0.1–100 ng/mL α-GalCer. (**C**) Mean division score of NKT cells after co-culture with splenocytes loaded with 0.1–100 ng/mL α-GalCer. All data displayed in graphs correspond to mean +/− SEM of 3 biological replicates. Statistical significance was determined using student’s t test or one-way ANOVA followed by Bonferroni tests. P values as follows: *p < 0.05, **p < 0.001, and ***p < 0.0001. Flow cytometry gating strategy outlined in Materials and Methods.

To address the caveat of NK1.1 downregulation in the bulk experiments, and to assess whether the observed phenomenon is impacted by the strength of antigenic stimulation, we titrated the amount of α-GalCer used to stimulate FACS-isolated stage 2 and stage 3 NKT cells. Stage 2 NKT cells significantly expand at lower levels of α-GalCer stimulation (1 ng/mL) as indicated by an increased total NKT count (p < 0.001, Fig. 6A), fold expansion (16.2-fold, p < 0.05, Fig. 6B), and mean division score (3.5, p < 0.001, Fig. 6C) compared to vehicle-stimulated stage 2 NKT cells. Conversely, stage 3 NKT cells do not significantly expand until 10 ng/mL of α-GalCer as demonstrated by an increased total NKT count (p < 0.001, Fig. 6A), fold expansion (16.5-fold, p < 0.001, Fig. 6B), and mean division score (4.8, p < 0.001, Fig. 6C) compared to vehicle-stimulated stage 3 NKT cells. Importantly, at 10 and 100 ng/mL of α-GalCer, stage 2 NKT cells expand significantly more than stage 3 NKT cells – resulting in significantly higher total NKT cell count (black asterisks, p < 0.0001, Fig. 6A) and fold expansion (p < 0.0001, Fig. 6B). CD28 blockade significantly reduces the expansion of stage 2 NKT cells at 1, 10, and 100 ng/mL as demonstrated by reduced total NKT count (red asterisks, Fig. 6A), fold expansion (Fig. 6B), and mean division score (Fig. 6C). Similarly, CD28 blockade significantly reduces the expansion of stage 3 NKT cells at 10 and 100 ng/mL as demonstrated by reduced total NKT count (red asterisks, Fig. 6A), fold expansion (p < 0.0001, Fig. 6B), and mean division score (p < 0.0001, Fig. 6C). While CD28 blockade precluded the expansion of stage 3 NKT cells, stage 2 NKT cells expanded at 10 and 100 ng/mL despite CD28 blockade – as indicated by increased fold expansion (Fig. 6B) and mean division score (Fig. 6C). Collectively, these results confirm that stage 3 NKT cells are more sensitive to CD28 blockade than stage 2 NKT cells.

### CD28 blockade inhibits NKT cell cytokine production

**Figure 7:**
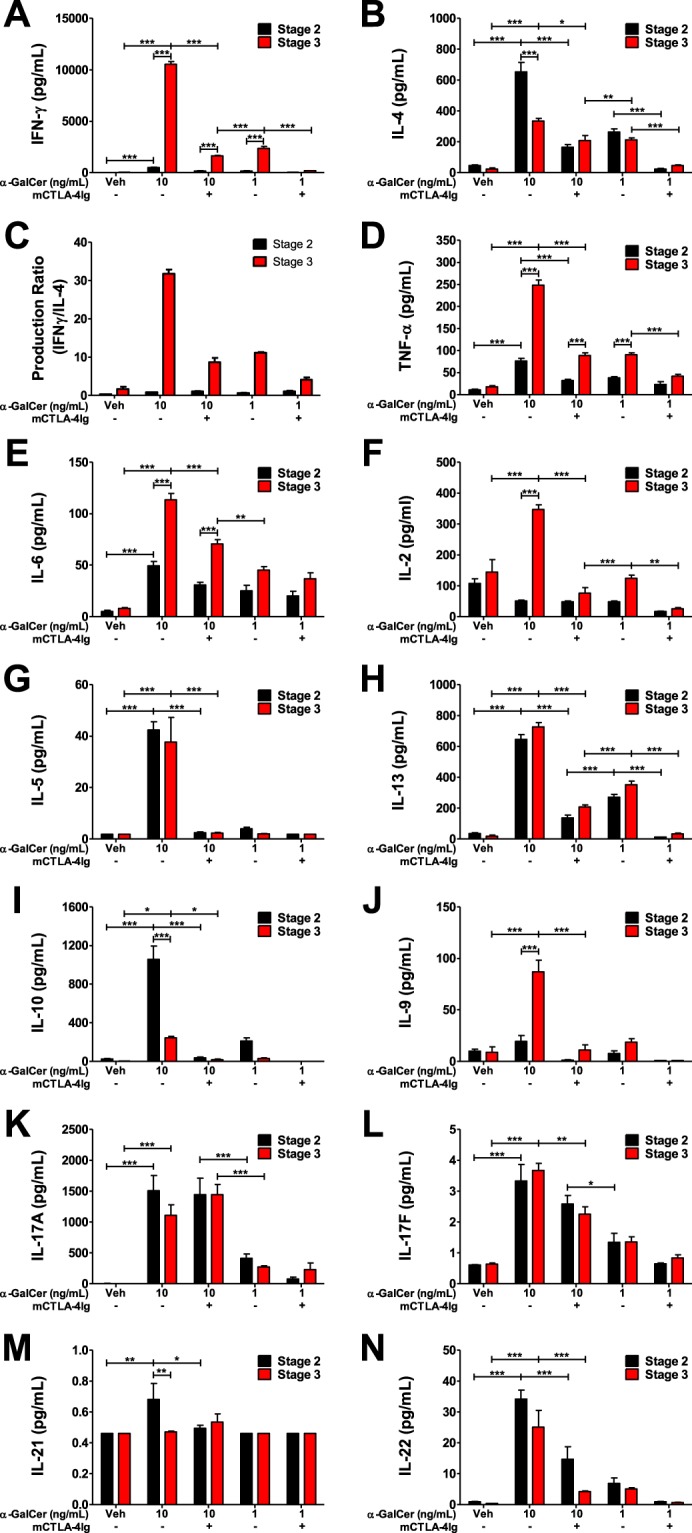
CD28 blockade inhibits NKT cell cytokine production. NKT cells were sorted as α-GalCer:CD1d Tetramer+ TCRβInt/+ CD24− CD44 + and NK1.1−(Stage 2) or NK1.1 + (Stage 3). Sorted NKT cells were co-cultured with splenocytes pre-loaded with vehicle or 1 or 10 ng/mL α-GalCer in the presence of media or CTLA-4Ig for 72 hours. Culture supernatants were analyzed for production of (**A**) IFN-γ, (**B**) IL-4, (**D**) TNF-α, (**E**) IL-6, (**F**) IL-2, (**G**) IL-5, (**H**) IL-13, (**I**) IL-10, (**J**) IL-9, (**K**) IL-17A, (**L**) IL-17F, (**M**) IL-21, and (**N**) IL-22. (**C**) Ratio of IFN-γ to IL-4 production by stage 2 and stage 3 NKT cells. All data displayed in graphs correspond to mean +/− SEM of 3 biological replicates. Statistical significance was determined using one-way ANOVA followed by Bonferroni tests. P values as follows: *p < 0.05, **p < 0.001, and ***p < 0.0001. Flow cytometry gating strategy outlined in Materials and Methods.

Given that expansion is only one outcome of NKT cell activation, we sought to determine additional functional differences between purified NKT cell subsets. Therefore, we assessed cytokine production by FACS-isolated stage 2 and stage 3 NKT cells after stimulation by splenocytes loaded with 1 and 10 ng/mL α-GalCer. At 10 ng/mL α-GalCer, stage 3 NKT cells produce significantly more T_H_1-associated cytokines such as IFN-γ (p < 0.0001, Fig. 7A), TNF-α (p < 0.0001, Fig. 7D), IL-6 (p < 0.0001, Fig. 7E), IL-2 (p < 0.0001, Fig. 7F), and IL-9 (p < 0.0001, Fig. 7J) whereas stage 2 NKT cells produced significantly more IL-4 (p < 0.0001, Fig. 7B), IL-10 (p < 0.0001, Fig. 7B), and IL-21 (p < 0.001, Fig. 7B). However, there was significantly cross production of some cytokines (such as IL-4 by stage 3 NKT cells, Fig. 7B) and IL-5 (Fig. 7G), IL-13 (Fig. 7H), IL-17A (Fig. 7K), IL-17F (Fig. 7L), and IL-22 (Fig. 7N) were equally produced by stage 2 and stage 3 NKT cells. Cytokine production was significantly reduced by antigen concentration and CD28 blockade (except for IL-17A, Fig. 7K). At 10 ng/mL of α-GalCer, stage 3 NKT cells produced significantly more IFN-γ than IL-4 as indicated by an IFN-γ/IL-4 ratio of 30 (Fig. 7C). This ratio was reduced to 10 by CD28 blockade (Fig. 7C) due to inhibition of IFN-γ production by 84.5% (Fig. 7A), but only a 37.7% reduction in IL-4 production (Fig. 7B). Therefore, although CD28 blockade reduces all cytokine production, stage 3-specific cytokines, namely IFN-γ, were more significantly reduced.

In this study we provide evidence demonstrating that distinct subsets of type 1 NKT cells have differential costimulatory requirements. We show that thymic NKT cell activation can be assessed *ex vivo* using a sensitive fluorometric proliferation assay. Our data suggest that thymic NKT cells are not affected by CTLA-4 signaling but require CD28 signals. Further stratification of NKT cell subpopulations using NK1.1 and PLZF expression reveals stage 3 NKT cells expand less efficiently than stage 2 NKT cells. CD28 blockade decreases the number of stage 3 NKT cells recruited to division after stimulation and decreases the proliferative capacity of both stage 2 and stage 3 NKT cells. Concordantly, CD28 expression is increased on dividing cells, but not undivided cells. Additionally, we found that PLZF expression changes with stimulation with the formation of a NK1.1-, PLZF-Lo population, and that PLZF is upregulated by TCR signaling prior to division. One caveat of the bulk experiments is that NK1.1 decreases following activation. Thus, ideal experiments would include NKT subset specific markers that do not change following stimulation, as well as the isolation of purified populations using FACS. Stimulation of FACS-purified stage 2 and 3 NKT cells with a range of antigen concentrations demonstrated that, although activated stage 3 NKT cells lose NK1.1 expression, stage 3 NKT cells expand less than stage 2 NKT cells and stage 2 NKT cells can respond to lower antigen concentrations. Finally, we found that cytokine production is negatively impacted by CD28 blockade and that stage 3 production of IFN-γ is more significantly reduced by CD28 blockade than production of IL-4. Collectively, these results indicate that NKT cell subpopulations respond differently to antigenic stimulation and have differential requirements for costimulation.

## Materials and Methods

### Mice

CD45.2 + C57BL/6 mice were purchased from The Jackson Laboratory. CD45.1 + CD45.2 + F1 C57BL/6 mice were generated in house by breeding Taconic CD45.2 mice to Jackson CD45.1 mice. All mice were housed under specific pathogen free conditions at the University of Maryland and all experiments were performed in accordance with procedures approved by the University of Maryland School of Medicine animal care and use committee. In this study, all mice were female. For the ex vivo experiments, one-four mice were used for splenocyte feeders and three biological thymocyte replicates were used for all figures with each biological replicate composed of 3 mice. For the in vivo experiment, mice were divided into 4 groups of 3 mice each. Mice received vehicle or 50 μg CTLA-4Ig IP followed by IV vehicle or 2 μg α-GalCer at least 1 hour later. Mice were harvested 48 hours later and thymic NKT cell activation was assessed by flow cytometry.

### Splenocyte antigen loading

Spleens were harvested from CD45.1 + CD45.2+ mice, processed into single cell suspensions using 70μm cell strainers. Red blood cells were lysed using ACK (Ammonium-Chloride-Potassium) lysis buffer and washed twice in NKT buffer (1 × DPBS without Ca or Mg), 2% FBS, 0.02% sodium azide)^34^. For antigen loading, splenocytes were resuspended at 3 × 10^6^ cells/mL in complete media with the antigen or vehicle control and plated 1 mL/well in a 48-well cell-culture plate. α-GalCer (Axxora, LLC) was used at concentrations of 0.1, 1, 10, and 100 ng/mL and the vehicle control (DMSO) was diluted in a similar manner to 10 or 100 ng/mL α-GalCer based on the top antigen concentration in the experiment. The plated cells were incubated in a 37 C, 5% CO_2_ incubator overnight. Antigen-loaded splenocytes were harvested and washed once with 10 mL complete media.

### Thymic NKT cell enrichment and purification

Thymi were harvested from CD45.2+ mice, processed into single cell suspensions using 70 μm cell strainers and washed once in 5 mL NKT buffer/thymus. For NKT enrichment, thymocytes were resuspended at 5 × 10^7^ cells/mL in 2% FBS/PBS. 2.5 × 10^8^ thymocytes were used per depletion and yielded approximately 1 × 10^7^ enriched cells. For the depletion, thymocytes were incubated with α-CD8 (clone 53–6.7, BioLegend at 5 μg/mL) and α-CD24 (clone M1/69, BioLegend at 10 μg/mL) for 15 minutes. 375 μL of sheep α-rat IgG Dynabeads (Thermo Fisher) were used per 5 × 10^7^ cells. After a 15-minute antibody incubation, thymocytes were washed in 10x volume of 2%FBS/PBS and resuspended in the initial volume. Antibody-labeled thymocytes were transferred into washed beads and incubated on a rotator for 20 minutes. Bead-bound thymocytes were removed by placing the mixture on a magnet and collecting the NKT-enriched supernatant. Supernatant was further enriched by placing the cell suspension in a fresh tube on the magnet to remove minor bead contaminants before being transferred to a 15 mL conical tube. Beads were washed once with 5 mL 2% FBS/PBS and the magnet process was repeated to isolate bead-free cells in the supernatant. The enriched NKT cell fraction in the supernatant was collected. For FACS-purification, enriched NKT cells were resuspended in staining buffer with FcBlock (BioLegend, clone 93) and incubated at room temperature for 15 minutes. Surface stain mixture, including PerCP-Cy5.5-TCRβ (BioLegend, clone H57–597), PE-CD24 (BioLegend, clone M1/69), APC-CD44 (BioLegend, clone IM7), PE-Cy7-NK1.1 (BioLegend, clone PK136), BV421-PBS-57:CD1d Tetramer (NIH Tetramer Core Facility), and Near-IR fixable live-dead (Thermo Fisher), was added and incubated at 4 C for 1.5 hours. Stained samples were washed three times prior to purification using the BD Aria II (gating strategy after Live/Dead displayed in Fig. 6A). Enriched and FACS-purified NKT cells were immediately loaded with proliferation dye.

### Proliferation dye loading

A circulating water bath was preheated to 37 C. The proliferation dye CFSE (Thermo Fisher) was diluted in 0.5% FBS/PBS. Enriched NKT cells were resuspended in 1.5 mL of CFSE by inverting tube and vortexing on medium speed. Cells were incubated in the water bath for 10 minutes and mixed twice by inversion. Then the cells were immediately transferred onto ice and an equal volume of FBS was mixed in by inversion. Cells were washed once with 5 mL 0.5% FBS/PBS and once with complete media.

### NKT and splenocytes co-culture

#### Enriched NKT cells

After counting, 1.2 × 10^6^ NKT cells were plated in 500 μL of complete media in a 48-well plate and pre-incubated with media, α-CTLA-4 (BD, clone UC10-4F10-11, 20 μg/mL), or isotype control (BD Pharmingen, clone B81-3, 20 μg/mL) for 1 hour at 37 C. Simultaneously, splenocytes were resuspended at 2.4 × 10^6^ cells/mL in complete media and pre-incubated with media or mCTLA-4-Ig (20 μg/mL) for 1 hour at 37 C. After pre-incubation, 1.2 × 10^6^ splenocytes in 500 μL were added to the wells containing NKT cells. Secondary antibody (α-hamster IgG, BioLegend, clone Poly4055, 1 μg/mL) was added to indicated wells. Note that enriched NKT cells must be plated with both vehicle-loaded splenocytes and α-GalCer-loaded splenocytes. The co-culture was placed in a 37 C, 5% CO_2_ incubator for 72 hours.

#### Purified NKT cells

1 × 10^4^ NKT cells were plated in 100 μL of complete media in a 96-well plate. Splenocytes were resuspended at 3 × 10^6^ cells/mL in complete media and pre-incubated with media or mCTLA-4-Ig (20 μg/mL) for 1 hour at 37 C. After pre-incubation, 3 × 10^5^ splenocytes in 100 μL were added to the wells containing NKT cells. Note that purified NKT cells were plated with vehicle-loaded splenocytes and splenocytes loaded with 0.1–100 ng/mL α-GalCer. The co-culture was placed in a 37 C, 5% CO_2_ incubator for 72 hours.

### Flow cytometry analysis

Day 0 samples, including pre- and post-enrichment thymocytes were surface stained to assess enrichment efficiency. Leftover splenocytes were used for the unstained and live/dead compensation samples and leftover thymocytes for the proliferation dye compensation sample. At 72 hours, the co-culture was harvested for analysis. Supernatant was removed and stored at −20C. Cells were resuspended in staining buffer and transferred to 15 mL conical tubes. Harvested cells were resuspended in 500 μL and counted. 300 μL and 200 μL of cells were transferred to the wells of a 96-well plate for intracellular (ICS) and surface staining respectively. For surface staining, cells were resuspended in staining buffer with FcBlock (BioLegend, clone 93) and incubated at room temperature for 15 minutes. Surface stain mixture, including BV711-CD45.1 (BioLegend, clone A20), BV570-CD45.2 (BioLegend, clone 104), PerCP-Cy5.5-TCRβ (BioLegend, clone H57–597), PE-CD8β (Thermo Fisher, clone H35-17.2), BV605-CD24 (BioLegend, clone M1/69), APC-CD69 (BioLegend, clone H1.2f3), APC- or PE-CD28 (BioLegend, clone 37.51), BV650-CD44 (BioLegend, clone IM7), PE-Cy7-NK1.1 (BioLegend, clone PK136), BV421-Unloaded or PBS-57:CD1d Tetramer (NIH Tetramer Core Facility), and Near-IR fixable live-dead (Thermo Fisher), was added to each well and incubated at 4 C for 1.5 hours. Surface stained cells were washed twice with 200 μL of staining buffer and resuspended in 200 μL staining buffer. For ICS, cells were fixed in BD cytofix/cytoperm for 20 minutes on ice followed by Thermo Fisher FoxP3 fix/perm for 2 hours on ice and then permeabilized by 2 washes with Thermo Fisher FoxP3 perm wash. ICS samples were resuspended in FcBlock in perm wash for 10 minutes on ice and then ICS stain mixture, including AF488- or PE-PLZF (Thermo Fisher, clone Mags.21f7), APC-RORγt (Thermo Fisher, clone Afkjs-9), and PE-Ki67 (Thermo Fisher, clone SolA15), was added to each well and incubated for 1 hour on ice. ICS samples were washed twice with perm wash and once with staining buffer then resuspended in 150 μL staining buffer. All samples were analyzed on the UMGCCC Flow Cytometry Core Cytek Aurora and data analysis was performed using FCS Express 6 Flow Research Edition by de Novo Software. The gating strategy was as follows: Singlets were selected using a FSC-H x FSC-A plot, live cells selected using a FSC-A x Live/Dead plot, CD45.2 single positive cells were gated using a CD45.1 x CD45.2 plot, α-GalCer tetramer, TCRβ double positive cells were selected using a α-GalCer tetramer x TCRβ plot. NKT cell expression of NK1.1, CD44, CFSE, CD28, CD69, PLZF, and RORγt were examined using dot plots and/or histograms. To determine precursor proliferation, NKT cell populations were stratified by CFSE fluorescence and division bins were created based on intensity peaks. Gate percentages were converted into absolute cell counts in Microsoft Excel and the number of precursor cells was determined by dividing by 2^i^ where i equals the number of divisions. The mean division number was calculated by excluding undivided precursors and summing all the divided precursors multiplied by their division bin and dividing by the total number of precursors. GraphPad Prism was used to prepare graphs and perform statistical analyses.

## Supplementary information

Supplementary Figure 1.

## Data Availability

The datasets generated during and/or analyzed during the current study are available from the corresponding author on reasonable request.
