# Supplementary material for: Thymic resident NKT cell subsets show differential requirements for CD28 co-stimulation during antigenic activation
Source: Sci Rep. 2020 May 19;10:8218. doi: 10.1038/s41598-020-65129-3 (PMC7237672; doi:10.1038/s41598-020-65129-3)
Supplement: Supplementary file 1 — Supplementary Figure 1. [file 41598_2020_65129_MOESM1_ESM.pdf]

Supplementary Information

Thymic resident NKT cell subsets show differential requirements for CD28 co-stimulation during antigenic activation

Susannah C. Shissler, Nevil J. Singh, Tonya J. Webb

Department of Microbiology and Immunology and the Marlene and Stewart Greenebaum Comprehensive Cancer Center, University of Maryland School of Medicine, Baltimore, MD 21201

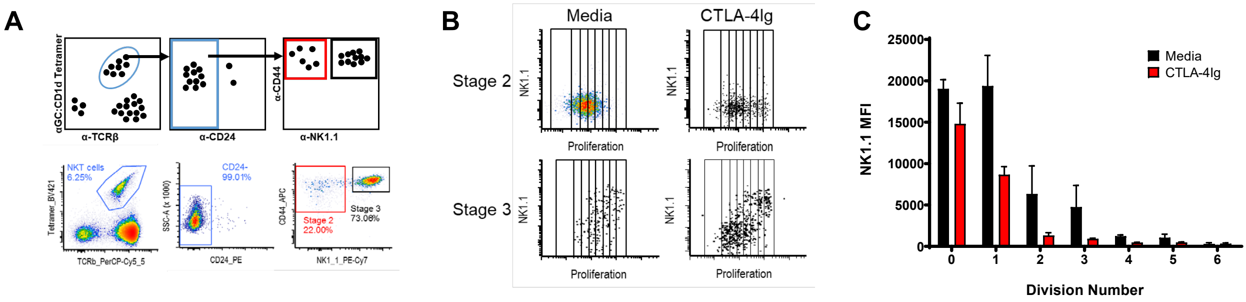

Supplementary Figure 1. Stage 3 NKT cells downregulate NK1.1 after activation.(A) NKT cells were sorted as α-GalCer:CD1d tetramer+ TCRβInt/+ CD24- CD44+ and NK1.1-(Stage 2) or NK1.1+ (Stage 3). Sorted NKT cells were co-cultured with splenocytes pre-loaded with vehicle or α-GalCer in the presence of media or CTLA-4Ig for 72 hours. (B) Representative plots of NKT cell division and expression of NK1.1 after co-culture with splenocytes loaded with 1ng/mL α-GalCer. (C) Mean fluorescence intensity (MFI) of NK1.1 on stage 3 NKT cells during successive rounds of division after co-culture with splenocytes loaded with 1ng/mL α-GalCer.
